# Does children’s fear matter? Evaluating children’s positions in Finnish court decisions on stalking

**DOI:** 10.1002/bsl.2590

**Published:** 2022-09-08

**Authors:** Sanna Koulu, Anna Nikupeteri, Merja Laitinen, Mirva Lohiniva‐Kerkelä

**Affiliations:** ^1^ Faculty of Social Sciences University of Lapland Rovaniemi Finland; ^2^ Faculty of Law University of Lapland Rovaniemi Finland

**Keywords:** children, court decision, discourse analysis, fear, position, stalking

## Abstract

Children are at particular risk when one parent is targeted by the other parent's stalking behaviors post‐separation. In this article, we explore how court decisions position children when assessing fear, distress, and unlawfulness in cases of parental stalking. The data comprised 127 court decisions on stalking that involved a relationship (dating, cohabitation, or marriage), separation/divorce, and one or more children. Using discourse analysis, we identified four categories in how children were positioned: (1) children relegated to the background, (2) children’s involvement recounted as part of the facts, (3) children’s involvement assessed as relevant because it affected the parent, and (4) children as agents or victims in their own right. The findings highlight a significant risk of losing sight of children when the focus is on parents, and our concern is that this may also contribute to children not receiving the support they need.

## 1 INTRODUCTION

Knowledge about stalking after the breakdown of a relationship has increased over the last 2 decades. Several studies have shown that children are at particular risk when one parent is targeted by the other parent's stalking behaviors post‐separation. Perpetrators often involve or even exploit children through their stalking behavior; children can be the targets of stalking or can be subjected to threatening behaviors aimed at the other parent or directly at themselves (Løkkegaard et al., 2019; Nikupeteri & Laitinen, 2015; Zeoli et al., 2013). Although separation or divorce creates physical distance, the use of technological devices and online media can make the stalking parent “omnipresent” in children’s lives (Dragiewicz et al., 2021; Khader & Chan, 2020; Markwick et al., 2019; Nikupeteri et al., 2021). Moreover, when there are children in common, custody issues and contact arrangements create an arena for the perpetrator to harass the ex‐partner and use these as reasons for establishing contact frequently (Bruno, 2015; Humphreys et al., 2019; Zeoli et al., 2013).

Stalking creates an atmosphere of fear and feelings of insecurity for children (Nikupeteri & Laitinen, 2015). According to Elklit et al. (2019), the outcomes of parental stalking for children involve complex, multiple‐event, and continuous trauma. In their study, which involved 57 children, 56% met the diagnostic criteria for post‐traumatic stress disorder. However, although children are victimized by stalking in several ways, they can also take multiple agent positions with regard to their parental and family relationships, wider social networks, cultural and moral practices, and professional encounters when shadowed by stalking (Laitinen & Nikupeteri, 2020).

Although there is no universal definition of stalking that has been adopted in legal and research arenas, the definition of stalking victimization typically includes two elements: (1) the course of conduct exhibited by the perpetrator and (2) feelings of fear by the victim (Fissel et al., 2020). In stalking, fear plays a crucial role, as fear, as a victim's emotional response to the perpetrator's behavior, is often involved in stalking criminalization (Dietz & Martin, 2007; Owens, 2016; Reyns & Englebrecht, 2013; van der Aa, 2018). In some jurisdictions, the legislation specifies that the victim must have been frightened by the stalking, while others require that stalking would have caused a reasonable person to experience fear (e.g., Fox et al., 2011). However, the requirement for fear varies, and some stalking statutes and definitions may use other comparable terms to describe the emotional response, such as “distress.” Often, the perpetrator's course of conduct is also assessed in terms of intentionality and unlawfulness (e.g., Logan & Walker, 2017). The nature of stalking as a repeated action and the dynamic nature of fear, which may change over time, create challenges in assessing victims' fear and distress (Reyns & Englebrecht, 2013). Even when children are not the primary victims of stalking, their sense of fear and distress is further accentuated by the power imbalance between them and the parent, as well as their dependence on adults for daily care (see Øverlien, 2013). The perpetrating parent can also deliberately appear as the “admirable,” “caring,” or “concerned” parent in ways that obscure their abusive behavior from professionals or communities (Katz et al., 2020). In some countries, antistalking laws give special attention to the vulnerability of the victim, for example, whether the victim is a child, which may affect the sentencing (van der Aa, 2018).

In this article, we examine how children’s positions were perceived in Finnish court decisions on stalking. Finland criminalized stalking in 2014, and court decisions from the years following the criminalization offer rich data on how the law understands and interprets children’s positions in cases of parental stalking. Finland's criminalization highlights several key elements of stalking, such as fear and distress, and it also explicitly requires the perpetrator's course of conduct to be unlawful. We approached the court decisions by examining how unlawfulness, fear, and distress were assessed when children were involved. Our research question was as follows: *How do court decisions position children when assessing fear*, *distress*, *and unlawfulness in cases of parental stalking?* We utilized discourse analysis, which enabled us to approach the court decisions as a social practice (Fairclough, 2001). This article contributes to both academic and professional discussions by raising awareness about children’s experiences and agency in parental stalking, which may further enable them to receive the support they need when they are exposed to parental stalking.

## 2 DATA AND METHODS

### 2.1 Court decisions on stalking

This article is based on a research project called Children’s Knowing Agency in Private, Multiprofessional and Societal Settings – the Case of Parental Stalking, wherein we explored children’s and young people's experiences and agency in parental stalking. The research project was approved by the Research Ethics Committee of the University of Lapland, and throughout the research process, we followed the ethical guidelines of the Finnish National Board on Research Integrity (2019). This article draws on uniquely comprehensive data based on Finnish court decisions on stalking. Our full data set comprises all court decisions on stalking given in the first 4 years since the criminalization of stalking has been in force (2014–2017); the cases were collected from all 27 Finnish district courts. In an unofficial translation, the relevant provision in the Finnish Criminal Code (chapter 25, Section 7a) stipulates the following:

a person who repeatedly threatens, observes, contacts or in another comparable manner unjustifiably stalks another so that this is conducive towards instilling fear or anxiety in the person being stalked, shall, unless an equally or a more severe penalty is provided elsewhere in law for the act, be sentenced for *stalking* to a fine or to imprisonment for at most two years.

Altogether, there were 419 decisions on stalking during the 4‐year period, covering a wide variety of situations. In this article, we focused on the decisions (*n* = 127) involving a relationship (dating, cohabitation, or marriage) between parents or a parent and another person, separation or divorce, and one or more children. Based on the information contained in the decisions, in most decisions, the primary victim was the ex‐partner (*n* = 124), while in the rest (*n* = 3), the primary victim was either a child or the ex‐partner's new partner. All the ex‐partner relationships in these decisions were heterosexual; a total of 116 cases involved a male ex‐partner stalking a female victim, while in eight cases, the ex‐partner perpetrator was female and the victim male. The rest were two cases of a female perpetrator stalking a female victim and one case of a male perpetrator stalking a male victim. The exact nature of the charges varied. In some cases, the stalking charge was the only one, whereas in others, the proceedings also covered other charges, either in relation to the same course of conduct or unrelated criminal offenses. Children's exposure to stalking varied broadly in the cases, and even in cases in which they were directly affected by it, they were relatively rarely recognized as injured parties.

### 2.2 Data analysis

The data analysis was content‐oriented, beginning with a reading of all case files related to stalking and coding them in an Excel spreadsheet. The coding included the basic information of each case, for example, duration of stalking, most relevant charges, court's decision on sentencing, and children’s possible involvement. We also numbered the decisions so that we could refer to them anonymously. This first phase was aimed at recognizing cases with post‐separation parental stalking. The most common relationships in the decisions were between children and their legal parents, but there were also some cases where the defendant was a step‐parent or a parent's new partner who had no relationship with the children.

The second phase involved a closer reading of the material involving parental stalking and children and then tabulating, in another Excel spreadsheet, the following thematic codes: acts of stalking, children’s relationship with the perpetrator and how stalking appeared in and affected children’s everyday lives, contacts with authorities, whether children’s experience were considered in the court's reasoning, and general relevant points concerning the stalking behavior and judicial process. The coding conducted in this phase highlighted the various scenarios in which children were involved in cases of parental stalking as well as the somewhat haphazard responses they received in judicial and service system processes.

In the third phase, we read the cases involving parental stalking and children by examining the discourses present in the decisions. Discourse analysis enabled us to explore the meanings produced by language use and interaction, the contexts and processes of these meanings, and the judicial practices connected to these meanings (Fairclough, 2001). We were interested in how children were positioned in the courts' evaluation of the unlawfulness, fear, and distress caused by stalking. We paid attention to what was going on in the cases, for example, activity, events, purpose, and topic (*contents*), who was involved (*subjects*), in what relations (*relations*), and what role language played in the decisions in the context of legal decision‐making (*connections*) (Fairclough, 2001). We then interpreted the situational contexts in the court decisions from the perspective of the positions that were constructed for children. We identified four categories in terms of how children were positioned: (1) *children relegated to the background* (*n* = 54), (2) *children’s involvement recounted as part of the facts* (*n* = 32), (3) *children’s involvement assessed as relevant because it affected the parent* (*n* = 27), and (4) *children as agents or victims in their own right* (*n* = 14).

The categories were not mutually exclusive or definitive. First, the decisions were categorized based on the most characteristic features of the decision; even a single decision may have included features of different categories, for example, in assessing different charges related to the same events (see also Fox et al., 2011). Second, the data set included cases where the same parties were involved in more than one set of proceedings, as the stalking continued after the first judgment (a total of 15 cases concerning five sets of parties), and the decisions often differed from each other in how children’s positions were constructed. For instance, sometimes the earliest decision may have barely mentioned the child, while a later decision depicted the child as an agent or victim in their own right.

## 3 CHILDREN’S POSITIONS IN COURT DECISIONS ON STALKING

In this section, we outlined the four categories that we identified in the case law as relevant to children’s positions and looked more closely at the courts' reasoning in each category. Table 1 summarizes the characteristics of the categories, children’s roles, and typical features in the assessment of the elements of the crime.

**TABLE 1:** Children's positions in court decisions on parental stalking

Category	Characteristics	Children's roles	Assessment of the elements of the crime
Children relegated to the background	‐ Children are mentioned, but their involvement is not relevant for the facts of the case or the elements of the crime	‐ Children as objects of parental communication	‐ Court does not mention children at all, or mentions children only in passing
	‐ The defendant maintains that they had been in contact about matters concerning the children and that the contact was thus justified	‐ Children as bystanders	‐ Facts and elements of the crime are assessed only from the adult parties’ perspectives
Children's involvement recounted as part of the facts	‐ Children's involvement is expressed in factual terms or in recounting the facts of the case	‐ Children as means or tools of stalking	‐ Court mentions children factually, as involved in the events under consideration
		‐ Children as direct or indirect targets of threats or efforts to disparage the ex‐partner	‐ Children's involvement is considered in terms of the facts of the case, while the elements of the crime are assessed only from the adult parties’ perspectives
Children's involvement assessed as relevant because it affected the parent	‐ Consequences of stalking faced by children are acknowledged or included in the assessment of the parent's fear and distress	‐ Children's feelings instrumentalized in stalking	‐ Court considers the children’s involvement in the assessment of the elements of the crime
	‐ Children's exposure to stalking is seen as affecting the parents' motivations or experiences, the child–parent relationship, or raising the children	‐ Children as collateral/secondary victims	‐ Children's experience may exacerbate the adult's fear or distress, or may emphasize the unlawfulness of stalking
		‐ Children as someone to protect	‐ The importance of children may constitute a reason for the defendant's motivation or behavior
Children as agents or victims in their own right	‐ Children's involvement is described in a way that acknowledges their agency or subjective experience as relevant	‐ Children as victims/agents in their own right	‐ Court assesses the children’s feelings and experiences as relevant for its judgment
		‐ Children as an injured party beside the adult/parent	‐ At least some elements of the crime are assessed from the children’s perspective

We used anonymized excerpts from the court decisions when discussing the categories. All excerpts were from the courts' assessment of the evidence or the elements of the crime, unless otherwise noted. All excerpts were translated by the authors from the original language, Finnish, and were lightly edited to ensure anonymity and clarity. However, the following excerpts were not meant to enable a comprehensive analysis of the case law from a legal point of view, and we focused on discursively significant passages instead of examining decisions from the viewpoint of legal doctrine.

### 3.1 Children relegated to the background

The first category, children relegated to the background, was the most numerous in our data set, with a total of 54 decisions. The first key feature in these decisions was that children were mentioned in passing, but their involvement was not relevant to the facts of the case or the elements of the crime. These mentions were often very brief or appeared almost as an afterthought, with little consistency in how the children were brought up, by whom, and to what end. The following two cases were different from each other in that in the first case, both parties were the child's parents, while in the latter, the defendant was not the father. However, in both cases, the children’s involvement was mentioned as a side issue in describing the course of events or the people involved in the situations:

When [the injured party] went on a cruise with her partner and daughter, [the defendant] sent her a message that he might be there as well. […] [The defendant] made it clear that he knew where [the injured party] goes and what she is doing. The messages came in bursts sometimes several times in a day and [the defendant] was present the whole time. (#79)

The letter shows quite clearly that the defendant has been following [the injured party] e.g. as she has taken her daughter to school and driven to [area], has visited [the injured party’s] work place and in that letter called [the injured party] especially disparaging names and also disparaged [the injured party’s new partner]. (#269)

The second key feature in these cases was related to communication about children. The defendants often maintained that they had been in contact solely about matters concerning the children and that the contact was thus justified. The children’s matters were often understood as contact arrangements, visitations, and maintenance, among other issues related to dealing with the separation (see also Humphreys et al., 2019; Zeoli et al., 2013). Mostly, this communication was conducted through technological means, such as sending text messages or WhatsApp messages. Although technology enables separated parents' positive and effective communication regarding children’s issues and care, it can also be misused and provide additional means for perpetrators to abuse victims (Markwick et al., 2019; Nikupeteri et al., 2021). Besides technological means, the defendant may also have contacted the children physically. For example, in one case, the defendant visited the injured party's home—where the child was also present—to ask how the child was doing. The court did not find this argument plausible.

In many cases, the defendant's contact with the injured party, supposedly in the name of the children’s issues, had little to do with the children. In both of the following examples, the parties had strong disagreements, for example, concerning contact with the children that they had in common, and the defendant's messages constituted part of the continuum of stalking:

[The defendant’s] messages to [the injured party], presented as written evidence, hardly concerned the parties’ child and issues related to visitation with them. The number of messages is large. The messages were inappropriate in their content and they were often also sent at inappropriate times of the day. In part the messages were also threatening. (#14)

[The defendant] and [the injured party] have two children in common, and [the defendant] only met them rarely during the time period specified in the charges. The District Court considers it somewhat credible that [the defendant] aimed at dealing with issues related to visitation with the children via his conduct but that he chose the wrong course of action. (#375, from the reasoning on the sentence)

In the decisions in this category, the defendant's claims of only having been in touch regarding children’s matters were rarely investigated in depth, and the children’s views on contact were not considered. In some cases, children were mentioned only by one of the parties and not in the courts' reasoning even though the defendant's conduct often appeared quite intrusive. In the following excerpt, the defendant was in contact with the injured party via phone, published messages about the injured party and her private life on social media, and kept showing up near her workplace or apartment:

[The defendant] denied the charges. Even though [the defendant] partly acted in the manner described in the charges, it was not his intention to offend or stalk the [injured party]. The defendant was calling on matters related to his and [the injured party’s] children. (#331, the defendant’s reply)

However, this category also included decisions with careful legal reasoning, even if the parties’ children were only considered in the abstract. For instance, some courts carefully assessed the contents of the parties’ communication and distinguished between parenting issues and interpersonal topics. In the following excerpt, the court considered that the defendant's messages were mostly related to the parties’ prior relationship and targeted the ex‐partner, even though they did have children in common and there were mentions of them in the messages as well. The court noted that the communications by the defendant

were not of a kind where their purpose would have been to handle issues related to the custody of the children. The District Court notes that based on the preparatory works the purpose of the legislator has not been that repeated inappropriate communications would become legitimate just by adding isolated comments on children. (#56)

The children’s roles in the decisions in this category were rather passive. Children became objects of parental communication when a parent was in touch with the ex‐partner about children’s issues without giving any agency to the children on matters that affected them (e.g., Birnbaum & Saini, 2013; Bruno, 2015). Children were also sometimes perceived as bystanders of the individual acts the perpetrator had committed as part of stalking, even if the presented facts indicated that the child must have been affected by the behavior. For instance, the next excerpt described a case where the injured party and the defendant were in a relationship, and the injured party's teenage daughter was mentioned in passing in the court's reasoning:

The injured party experienced [the defendant’s earlier behavior] as extremely stressful and the injured party’s hands trembled every time she saw the defendant near her apartment. At times the injured party’s 17‐year‐old daughter was with the injured party. According to the injured party this kind of behavior caused feelings of fear and the situation was really rough. (#68)

In the decisions in this category, children were not considered in the assessment of unlawfulness or the fear and distress caused by stalking, even when they were mentioned. Stalking was considered a matter for the adults involved. Generally, the decisions in this category were sparse on legal reasoning. In several cases, the defendant had admitted the course of events or confessed to the crime, so legal reasoning was accordingly limited in scope. In other cases, there might have been multiple charges on unrelated offenses, so the bulk of the court's reasoning was related to other crimes. Nevertheless, it was typical of this category that the facts at hand, as well as the elements of the statutory definition of stalking—fear, distress, and unlawfulness—were assessed only from the adult parties’ perspectives and with little reference to the children’s experiences.

### 3.2 Children's involvement recounted as part of the facts

In the second category, children’s involvement became more visible as their presence was recounted as part of the facts of the case. The key feature characterizing the 32 decisions in this category was that children’s involvement in the cases was expressed in factual terms. However, decisions in this category were distinguished by a procedural approach, and there were no references to the children’s experiences or thoughts, even when they might have been directly impacted by stalking. Typically, children’s involvement was described narratively in reviewing the evidence before the court moved on to its reasoning on the elements of the crime.

In this category, children’s involvement was typically described in a neutral and technical manner. For example, children were mentioned when they had been present in physically abusive events; when the defendant had followed the injured party, who had been running errands with the children; or when the injured party had sought a restraining order to protect the children as well as themselves. In several cases, the court factually evaluated the defendant's claims, as illustrated in the next excerpt, where the defendant was in contact with both her ex‐partner and his new partner. The court considered the calls that the defendant claimed were made by her son in evaluating the violation of a restraining order:

[The defendant] has suggested that the caller may have been her [teenaged] son. [The injured party’s] testimony makes it clear that he did not have any contact with the defendant’s son after the divorce and that the defendant had specifically forbidden any contact between the son and [the injured party] and that the son knew [the injured party’s] phone number. Thus the District Court does not find the defendant’s account, that the caller would have been her son, credible. (#104)

In other cases, legal reasoning was more reflective. The following excerpt involves a case in which the defendant mainly used technological devices to contact his ex‐partner about the children, and the court assessed the problematic nature of technology and the importance of children’s experiences from the injured party's perspective:

In part the content of the messages was disruptive and offensive and some of the messages involved threats to [the injured party]. It is understandable that [the injured party] was not able to set a complete block on her phone, as she still had to be reachable for arranging matters concerning the children. (#249)

A key feature in this category was that although children were mentioned in the decisions, the texts lacked any acknowledgment of the children’s agency and subjectivity. Children were, in fact, often involved in stalking as the means or tools the perpetrator employed to get information concerning the ex‐partner (Nikupeteri & Laitinen, 2015). The perpetrators also used children as enablers or excuses to physically approach the ex‐partner. In the following two excerpts, the parties were divorced, but the defendant continued contacting the ex‐partner through the children:

In fact [the defendant] used the children to try and get information from them on what [the injured party] was doing and where she was or who was at her place. [The injured party] has to turn off the children’s phones when they are with her, as [the defendant] called them numerous times from morning to evening. (#183)

Even though he had an apartment of his own, [the defendant] came to [the injured party’s] home in a disruptive manner and tried to use their children to let him in [the injured party’s] apartment. […] Furthermore [the injured party] said that [the defendant] followed her in his car, and constantly tried to find out through the children where she was and where she was going. (#161)

Children were also involved as direct or indirect targets of the defendants' threats and efforts to disparage or harass the ex‐partner (also Nikupeteri et al., 2021; Toews & Bermea, 2017). The behavior included threats of violence and death or about committing suicide. Although such threats were sometimes expressed directly to the children themselves, the impact of the threats on their feelings was typically not considered in the decision. Instead, the approach was usually factual and narrative (“and then this happened, and then this, and then…”). In addition to ominous messages, such as threatening to strangle the other parent in a phone call to the child, the messages sent to the children often included insulting comments concerning the ex‐partner or false accusations concerning their competence and respectability as a parent. In the first of the following cases, the defendant kept involving her ex‐partner's new step‐daughter in the case, whereas in the second case, the defendant repeatedly disparaged his ex‐partner to the children after the divorce:

[The injured party #2] said that she experienced the situation as distressing. She had trouble sleeping because of it. [The injured party #2] was concerned about her own health because of the situation. [The injured party #2] did not understand what role she had in the divorce between [the injured party #1] and [the defendant], nor why the existence of her daughter kept turning up in the messages. The daughter was 17 years old and did not always dare to be at home alone because of the fear that the defendant would come visiting. (#103)

In the messages sent to the children [the defendant] used disparaging expressions to refer to [the injured party] and claimed that she was a liar. In part the messages were also coercive. (#309)

It is worth noting that although children’s involvement was recounted in the decisions, it often had no visible effect on the assessment of the elements of the crime. For instance, these decisions included cases where there were multiple mentions of children, as well as detailed descriptions of events that would have definitely impacted their lives; however, the defendant's course of conduct was considered justified in very succinct terms without any consideration of the effect of their actions on the children. In the following case, where the parties had shared custody of the children after their separation and the defendant kept appearing repeatedly in children’s daily venues, such as kindergarten and hobbies, the court did not consider the impact on the children.

The District Court finds that [the defendant] had the right to be present at the venues for the children’s hobbies and to take part in matters related to their shared custody. (#43)

Overall, the elements of the crime were assessed only from the adult parties’ perspectives, and the assessment made little reference to children’s experiences; however, legal reasoning was more diverse than that in the first category, where children were relegated to the background. The charges were often denied; thus, the courts needed to consider evidence more thoroughly than they would if the defendant had admitted to the crime. Several decisions included detailed descriptions of events, either based on hearing the parties or drawing on witness testimonies. In other cases where the same set of events led to multiple criminal charges, children may have been mentioned or their involvement described in charges related to another offense, such as menace or violation of a restraining order.

### 3.3 Children's involvement assessed as relevant because it affected the parent

The third category included a total of 28 decisions in which children’s exposure to stalking was acknowledged in relation to the elements of the crime, namely, as it affected the parents' experiences. While tension between the defendant's claims and the factual course of events was also present in several decisions in our previous categories, here the texts provide more references to emotional experiences and the relationship between a child and a parent.

In this category, the courts typically mentioned the consequences of stalking to children or included them in their assessment of the parent's fear and distress. The risk of children being harmed and in fear for their safety are often a concern for abused parents (Zeoli et al., 2013). Here, we want to mention two excerpts in which several criminal charges, including charges of physical abuse, were involved. In both cases, the courts described the injured parties’ fear for the children as part of the evidence of their own fear and distress:

Due to [the defendant’s] actions [the injured party] was very anxious and afraid. She was not able to live her life normally and did not dare to see her friends. Being outside her home made [the injured party] feel afraid. [The injured party] was forced to live in a shelter and did not always dare to take her child to daycare as she was afraid for both of them. (#110)

[The injured party] experienced the communications as distressing as well as threatening. The communications also stopped her from working as clients were not able to reach her. [The injured party] was also concerned about her children. (#85)

In other cases, the consequences of stalking were seen as pertaining to the relationship between the parent and the child or to raising the children. For instance, the courts sometimes considered the injured party's role as a parent as they evaluated the impact caused by stalking behavior:

It has been demonstrated on the basis of the medical opinion from the psychologist and witness testimonies that the injured party suffered from great and long‐lasting anxiety and fear. This conclusion is not affected by the fact that the injured party was able to function almost normally in employment and in the upbringing of children. (#41, from evaluation of damages)

[The injured party] said that [the defendant’s] communications caused her anxiety that then also affected her children. (#125)

The courts could also acknowledge the importance of children to the defendant's motivation even though they often still found the defendant guilty in these cases. While contact with children was also a recurring theme in earlier categories, here, the defendants' role as parents was typically described in a more detailed and thoughtful manner. Often, the courts also proceeded to evaluate the contents of the messages or communications from the defendant and assessed the defendants' motivations as to whether their actions were truly based on genuine interest in the children. This is noteworthy, as studies show that abusive parents do not always contribute to their children’s care and may instead ignore their needs (Cater & Forssell, 2014; Humphreys et al., 2019; Toews & Bermea, 2017). In some decisions, the courts' reasoning even condemned the defendants' lack of care toward their children more or less explicitly. The first excerpt shows a case in which the defendant had submitted false reports to child protection services, and the second case illustrates the harmfulness of the defendant's persistent stalking behavior toward the ex‐partner:

The court finds that the reports to the child protective services were manifestly ill‐founded and [the defendant] did not have any real concern about the children’s wellbeing, but that he instead made the reports with clear intention to harass. (#182).

The court finds it very blameworthy that [the defendant] did not stop stalking the injured party, even though he had already been sentenced several times for similar acts and in addition he must obviously have been aware that his conduct was extremely harmful to his own child. (#139)

Children's roles in this category often highlight their vulnerability. The decisions included cases where defendants had used their children’s feelings as a means to increase the primary victim's sense of fear or distress, essentially instrumentalizing those feelings in stalking behavior (also Beeble et al., 2007; Toews & Bermea, 2017). Often, the stalking behaviors experienced by the abused parent and children were profoundly interconnected, and they shared the context of fear and control (Humphreys et al., 2019). In other cases, children were depicted, especially by the injured party, as someone to be protected, someone to be afraid for, or as secondary victims. The following two excerpts are from cases in which the parties had separated after a rather short relationship and did not have children in common. In the first case, the defendant threatened the injured party's child in a text message, whereas in the second case, the injured party was afraid for the child's safety owing to the defendant's persistent behavior:

[The defendant] also threatened [the injured party’s] four‐year‐old daughter [name]. One possible criterion of the statutory definition of menace is that the injured party has justified reason to believe that his or her personal safety or that of someone else is in serious danger. The District Court finds that the threat to [the daughter] was intended by [the defendant] to threaten [the injured party] in particular. (#110, reasoning on a menace charge)

[The injured party] said that [the defendant’s] course of conduct was very distressing due to its persistence. Because of it she sought health care and had to take action to protect herself and the children e.g. by moving and by applying for a non‐disclosure order on her personal data. (#365)

In this category, children’s experiences of fear and distress are narrated by someone, for example, a parent or step‐parent; however, unlawfulness, fear, and distress are not evaluated from the children’s perspective. Instead, there can be a subtle disconnect between the parties’ understanding of the events and the courts' reasoning, where children can disappear from the text between recounting the evidence and legal reasoning. The legal reasoning in this category could be characterized as children’s involvement having an indirect effect on the elements of the crime. That is, their involvement, experiences, or rights matter in the assessment of one or more elements of the crime. However, these things are only relevant insofar as they affect a parent's feelings or conduct, and the experiences of the adults are centered. Children's involvement can exacerbate the parent's fear or distress, or affect the unlawfulness of stalking.

### 3.4 Children as agents or victims in their own right

Our fourth category is the smallest, with only 14 decisions. The distinguishing feature in this category is that in these decisions, children’s involvement is described in a way that acknowledges their agency and victimization as relevant with regard to the elements of the crime. That is, children’s fear and distress, as well as the unlawfulness of the defendant's course of conduct toward the children, were evaluated in themselves and not solely in light of how they affected the adults. The key characteristic of the cases in this category was that children were described both as agents and victims and that they were able to take multiple agent positions in their family relationships (Laitinen & Nikupeteri, 2020). Often, the children were also in their teens, that is, on average, older than those in the cases in other categories. The decisions spanned a wide variety of family arrangements, timeframes, and situations, and the cases had perhaps less in common with each other compared with those in other categories.

The children’s roles in these decisions partly mapped onto their standing in the legal proceedings. In several cases, children were involved as an injured party along with their parent, for instance, where the charges included a violation of a restraining order that protected both the child and parent. In other cases, children were the primary victims of either the stalking charge itself or of related offenses, such as menace. However, in addition, there were decisions in which children were acknowledged in their own right without having legal standing as the injured party. In these cases, the courts' legal reasoning regarding the unlawfulness of the defendant's course of conduct touched upon the fear or distress experienced by the children or positioned the children as having agency of their own. In effect, the courts utilized both a “rights perspective” and a “care perspective” in assessing stalking from the children’s point of view (Eriksson & Näsman, 2008). The decisions reflect the duality of children’s roles as vulnerable victims and as individuals with agency concerning their own lives. In the first case, a 15‐year‐old child was recognized as the injured party, and the court assessed the elements of the crime with regard to her experience, whereas in the second case, the court considered the children’s agency with regard to their age even though they did not have legal standing as injured parties themselves.

In her hearing [the injured party] said that [the defendant’s] course of conduct caused her anxiety and the anxiety affected her schoolwork. […] Some of the messages sent by [the defendant] were oppressive in tone, and in effect emphasized [the injured party’s] responsibility and even blame with regard to the functioning of social relationships. (#124)

In addition, the children were old enough that [the defendant] could have been in touch with them directly by e.g. e‐mail in addition to phone calls. Furthermore, the children had the opportunity to give [the defendant] their secret phone number as well, should they have wished to do so. (#229)

Several decisions in this category account for children’s involvement in legal reasoning and indicate that courts do find children’s perspectives meaningful. Some decisions were noteworthy in giving the children a literal voice in the text by including details about their views. Other decisions depicted children’s agency in relation to family members (Laitinen & Nikupeteri, 2020). Children appeared, for example, as individuals who could help protect the injured party or act against the perpetrator, as the following excerpt illustrates. In this case, the parties were separated, and the decision seemed to indicate that the children were already in their teens:

When heard in the District Court under an obligation to speak the truth, [the injured party] said that her youngest son was leaving for school one morning and called [the injured party] that ‘that one asshole is here again’. When [the injured party] asked who, the son answered ‘well who could it be’. [The injured party] asked her son to leave the place and called the police. The police went there and afterwards they reported to [the injured party] that [the defendant] had been taken away from the premises. (#214)

[The defendant’s conduct] caused [the injured party] fear, anxiety, shortness of breath, panic attacks, and difficulty sleeping among other things. She constantly had to resort to having one of her sons stay at her place at night and did not dare to go out alone at all anymore. (#214, from the injured party’s testimony)

Even when the children’s feelings and experiences were assessed as relevant to the court's reasoning, they were not necessarily a decisive factor. Children's views concerning their family relations may be disregarded even when they are very clear that they do not want to be in contact with the abusive parent (also Eriksson & Näsman, 2008; Øverlien, 2013). In one case, a child was targeted by his father's stalking behavior after his parents' separation, and the father was charged with dozens of violations of restraining orders and one charge of stalking. While the decision described the child's experience in detail, the father's motivation was not considered blameworthy:

[The child] described living in an atmosphere of constant alertness, fear and irritability as well as a state of stress, which is also supported by the public authorities' documentation. […] The District Court considers it evident based on [the defendant’s] testimony that his most factual motivation was to see his child, even though the child did not want to meet him. […] The motives for [the defendant’s] course of conduct were not unlawful or blameworthy […]. (#65)

The cases belonging to this category were quite diverse, so any conclusions from the legal reasoning in these decisions should be drawn carefully. In all cases, at least some elements of the crime were assessed from the child's perspective, but otherwise, there were noteworthy differences as well. Generally, two strands of reasoning presented themselves. First, in some decisions, children’s experiences were discussed, especially in relation to the fear and distress required by the statutory definition. In many of these decisions, the children were not yet in their teens. Second, in other decisions, the children’s experiences were relevant for considering the unlawfulness of the defendant's conduct, especially with regard to the defendant's stated wish for contact with them. Here, children’s agency tended to be the most visible in decisions where they were older teens or when the defendant was their step‐parent instead of a biological parent.

## 4 DISCUSSION

The four categories we distinguished from the data set show a variety of legal responses to children’s involvement in cases of parental stalking. In the first category, children were barely present in the background, whereas in the second and third categories, their presence was acknowledged either factually as part of a sequence of events or in more affective terms, as their presence affected the parents' experiences and motivations. In the fourth category, children were present in their own right, either as injured parties themselves or via decisions acknowledging their subjectivity and experience. While the decisions in the different categories were superficially similar, in that they all followed the structure set out in criminal and procedural legislation, several texts showed nuance in accounting for children’s views and experiences, as well as parent–child relationships. Taken together, these decisions provide a multifaceted picture of children’s positions in the assessment of the fear, distress, and unlawfulness of parental stalking. Moreover, they show that children also often feel fear in these cases, but courts rarely focus on their experiences, even though children can be seen as particularly vulnerable victims of stalking (see van der Aa, 2018).

There were several overarching themes in the decisions. First, the decisions highlighted the difficulties of post‐separation parenting in connection with stalking and abuse. A recurring theme throughout all categories included the defendants' claims of only wanting to be in contact with their children. In particular, the reasoning regarding the unlawfulness of the defendants' actions often highlighted the complexities of post‐separation family relations and considered the line between a parent's wish for contact based on sincere care for the children and the tactics of stalking and harassment to control the ex‐partner (Katz et al., 2020). In almost all cases in our data set, it was clear that the line was firmly crossed, but there were a few decisions in different categories that specifically included reasoning regarding unlawfulness and contact. In some cases, especially in the first category, the reasoning regarding post‐separation parenting and unlawfulness was more implicit than explicit in that the justifiable nature of being in contact because of children was a given.

Second, partly because of this complexity in post‐separation situations, the reasoning regarding unlawfulness often touched upon fear and distress as well, although the reasoning was often quite sparse, especially in the first category. Since the statutory definition of stalking requires culpability with regard to all elements of the crime, the prosecutors had to show that the defendants should have been aware that their actions were unlawful. This, in turn, may have been easier in cases where they were also in violation of a restraining order or were otherwise aware of the injured parties’ fear or distress. We found it especially noteworthy that many decisions, especially in the third and fourth categories, also commented on children’s involvement in these cases and even gave children a voice in describing the effect of the defendant's actions. This shows how courts can give children a role and an opportunity to voice their views in framing the family situation (see Heimer et al., 2018).

The third theme we noticed was that children were sometimes present in the decisions as part of the family rather than as individuals with views and thoughts of their own. For instance, the defendants' actions were often described as impacting the whole family or causing the family harm, and the effect of their actions on the children was not considered specifically. This perspective was present in the first category, where children were relegated to the background; however, family relationships were also given weight in the decisions in the second and third categories, and the fourth category was the most nuanced in accounting for children’s individuality and agency alongside their family relationships. When interventions target the family as an entity, there is a risk that individual children may not be identified and may end up missing the provision and protection they need (Heimer et al., 2018). This is worth noting, as many children experience symptoms of trauma following exposure to the stalking of their parent, and they need appropriate treatment (Elklit et al., 2019).

Using court decisions as a data set has its limitations. The main challenge was that the number of decisions given in Finland from 2014 to 2017 was limited; especially, the smaller categories had fewer than 30 decisions each. When the cases in each category also varied significantly, it was not possible to evaluate trends or commonalities in terms of how the stalking provision was applied in the first 4 years of legal practice. Moreover, several decisions were succinct and included limited information on the children. Although this tendency was the most pronounced in the first category, it appeared in the decisions in all categories. The categories do not fully map onto the everyday lives of the families, and knowing more of the circumstances behind the cases, for example, through pretrial materials, would have allowed us to assess the involvement of children better. Considering how brief and haphazard some of the mentions were, especially in the first category, it is possible that our full data set included decisions where children were literally invisible, that is, the parties’ children were not mentioned at all.

The context of criminal proceedings should also be noted in contextualizing our findings. The criminalization of stalking entered into force in Finland in 2014, and it meant that the prosecutors and courts had to determine exactly which acts constituted part of stalking and which were menace, violation of a restraining order, invasion of domestic premises, or harassing communications. As the criminalizations do have some overlap, in that the same course of events may fulfill the criteria of more than one statutory definition, the exact role of children may have depended on how the public prosecutor had chosen to press charges in each case. As some defendants had been charged with stalking repeatedly, often targeting the same person, we also had to consider how to analyze decisions that only covered part of the stalking behavior.

## 5 CONCLUSION

Overall, our analysis shows that children are often relegated to the background in court proceedings related to stalking and that children’s views or experiences are rarely acknowledged. Most decisions in our data set belonged to the first two categories, which rarely acknowledged children’s views or experiences, and in many cases, the courts' reasoning paid little attention to children, even when the parties themselves had brought them up in their testimonies. This may indicate that the structural constraints of the criminal justice system, with its crucial focus on legality and the right to fair trial, are not always able to make room for the complexities of victims' everyday lives.

Our study found that there is a significant risk of losing sight of children when the focus is on parents. The concern is that this may contribute to children not receiving the support they need when they are exposed to parental stalking. Children's vulnerability as victims and the importance of psychosocial counseling and support for them have also been highlighted internationally, for example, in the 2012 EU Victim Directive (2012/29/EU) and in connection to the Istanbul treaty (see van der Aa, 2018, and GREVIO, 2019, para 131). Thus, our study highlights the importance of paying attention to children in cases in which they are impacted by stalking behavior. Heimer et al. (2018) noted that when children are given the opportunity to voice their views on their own situation, it appears to affect both the protection and the provision offered to them in the context of social services. Finally, our findings emphasize the importance of incorporating both a rights perspective and a care perspective on children (Eriksson & Näsman, 2008) to safeguard children’s well‐being in cases of parental stalking. Our hope is that these strands of research can contribute to a heightened awareness of children’s agency and vulnerability in these complicated and multifaceted cases and help in understanding the implications of legal proceedings for children’s rights and well‐being more thoroughly in the future.

## CONFLICT OF INTEREST

The authors have no conflict of interest to declare.
